# Self-regulation of circumscribed brain activity modulates spatially selective and frequency specific connectivity of distributed resting state networks

**DOI:** 10.3389/fnbeh.2015.00181

**Published:** 2015-07-14

**Authors:** Mathias Vukelić, Alireza Gharabaghi

**Affiliations:** ^1^Division of Functional and Restorative Neurosurgery and Division of Translational Neurosurgery, Department of Neurosurgery, Eberhard Karls University TuebingenTuebingen, Germany; ^2^Neuroprosthetics Research Group, Werner Reichardt Centre for Integrative Neuroscience, Eberhard Karls University TuebingenTuebingen, Germany

**Keywords:** self-regulation of brain activity, neurofeedback, brain-computer interface, resting state networks, functional connectivity, corrected imaginary part of coherency, neuronal reorganization, Hebbian-like plasticity

## Abstract

The mechanisms of learning involved in brain self-regulation have still to be unveiled to exploit the full potential of this methodology for therapeutic interventions. This skill of volitionally changing brain activity presumably resembles motor skill learning which in turn is accompanied by plastic changes modulating resting state networks. Along these lines, we hypothesized that brain regulation and neurofeedback would similarly modify intrinsic networks at rest while presenting a distinct spatio-temporal pattern. High-resolution electroencephalography preceded and followed a single neurofeedback training intervention of modulating circumscribed sensorimotor low β-activity by kinesthetic motor imagery in eleven healthy participants. The participants were kept in the deliberative phase of skill acquisition with high demands for learning self-regulation through stepwise increases of task difficulty. By applying the corrected imaginary part of the coherency function, we observed increased functional connectivity of both the primary motor and the primary somatosensory cortex with their respective contralateral homologous cortices in the low β-frequency band which was self-regulated during feedback. At the same time, the primary motor cortex—but none of the surrounding cortical areas—showed connectivity to contralateral supplementary motor and dorsal premotor areas in the high β-band. Simultaneously, the neurofeedback target displayed a specific increase of functional connectivity with an ipsilateral fronto-parietal network in the α-band while presenting a de-coupling with contralateral primary and secondary sensorimotor areas in the very same frequency band. Brain self-regulation modifies resting state connections spatially selective to the neurofeedback target of the dominant hemisphere. These are anatomically distinct with regard to the cortico-cortical connectivity pattern and are functionally specific with regard to the time domain of coherent activity consistent with a Hebbian-like sharpening concept.

## Introduction

Brain-computer interfaces are currently being applied in neurofeedback training for a variety of brain-related pathological conditions to alleviate related symptoms (Wyckoff and Birbaumer, 2014). In such an environment, contingent feedback of the neuronal state is provided to enhance self-regulation of brain activity via operant conditioning. This neurofeedback training is expected to selectively induce use-dependent neuroplasticity for re-normalizing pathological brain activity and achieving behavioral gains (Daly and Wolpaw, 2008). Although variances of brain self-regulation could be attributed to different neuronal processes (Blankertz et al., 2010; Grosse-Wentrup et al., 2011; Halder et al., 2011; Vukelić et al., 2014; Vukelić and Gharabaghi, 2015), the underlying mechanisms of learning this skill still have to be uncovered to exploit the full potential of this technique for clinical application (Bauer and Gharabaghi, 2015a, b).

Due to its procedural nature and the involvement of the cortical-basal ganglia loop (Birbaumer et al., 2013), the skill of volitionally changing brain activity has been proposed to be comparable to implicit motor skill learning. Several neuroimaging studies revealed that a distributed network consisting of prefrontal, premotor, supplementary motor, primary sensorimotor, and parietal regions is recruited when acquired motor skills are executed (Hallett and Grafman, 1997; Halsband and Lange, 2006; Hardwick et al., 2013). What is more, resting state measurements, being unbiased by activity during any task, revealed that-particularly in fronto-parietal areas these networks were specifically modulated by previous motor skill *learning* but not by the motor *performance* (Albert et al., 2009). In addition, motor learning resulted in functionally distinct changes in subsequent intrinsic networks, revealing a distributed pattern of sensory and motor plasticity (Vahdat et al., 2011). These studies suggested that intrinsic resting state activity may reflect the processing of memory during consolidation, thereby resembling functional neuronal networks involved in skill learning (Albert et al., 2009).

In this context, we hypothesized that volitional modulation of brain activity modifies subsequent intrinsic networks similar to motor learning. Moreover, we expected these *resting state* networks to show a topographic distribution of synchronized cortical regions similar to that observed *during* neurofeedback training (Grosse-Wentrup et al., 2011; Halder et al., 2011; Vukelić et al., 2014; Vukelić and Gharabaghi, 2015) due to the cognitive demanding nature of brain self-regulation (Wander et al., 2013). On the basis of our previous findings during volitional brain control (Bauer et al., 2015; Vukelić et al., 2014; Vukelić and Gharabaghi, 2015) we went on to hypothesize that frequency-specific and spatially selective changes of functional connectivity occur and therefore applied a high-density electroencephalography study to capture connectivity patterns via the concept of the imaginary part of the coherency function (Nolte et al., 2004; Ewald et al., 2012). This approach has been applied in recent studies as a robust method to interfere functional connectivity (Martino et al., 2011; Dubovik et al., 2012; Westlake et al., 2012; Mottaz et al., 2014; Notturno et al., 2014).

## Materials and Methods

### Subjects

We recruited eleven healthy subjects (mean age = 25.83 ± 3.1 years, four female), all of them right-handed as assessed by the Edinburgh Handedness Inventory (Oldfield, 1971). Subjects gave their written informed consent before participation and received monetary compensation. The study protocol was approved by the local ethics committee of the Medical Faculty of the University of Tuebingen, Germany. The current data were collected as part of a larger research project investigating the neurophysiology of neurofeedback; whereas previous work analyzed the cortical physiology *during* neurofeedback training (Vukelić and Gharabaghi, 2015), this evaluation focused on the resting state networks *after* the interventions.

### Data Acquisition and Experimental Paradigm

All subjects were comfortably seated upright in a chair. High resolution scalp EEG potentials were recorded (BrainAmp, Brainproducts GmbH, Germany) from 128 positions according to the extended international 10–05 system, with active electrodes based on Ag/AgCl (actiCAP, Brainproducts GmbH, Germany). The left mastoid was used as common reference and grounded to AFz. All impedances were kept below 20 kΩ at the onset of each session. EEG data was digitized at 1 kHz, high-pass filtered with a time constant of 10 s and stored for off-line analysis (Brainvision, Brain Products GmbH, Germany).

Each subject was exposed to one neurofeedback training experiment, lasting 48 min, to acquire volitional control of regional low β-oscillations (16–22 Hz) induced by kinesthetic motor imagery of hand movements (right and left hand) which resulted in strong sensorimotor power fluctuations contralateral to movement imagination. The successful control of contralateral sensorimotor β-oscillations was translated into contingent neurofeedback. In order to reduce the impact of the feedback modality, participants received 24 min of haptic feedback (control of a hand orthosis which was attached to the right or left hand of the subjects) and 24 min of visual feedback (control of a cursor ball towards a selected target on a computer screen) in a randomized order. In order to balance for the impact of cerebral specialization, the subjects had to self-regulate either left (FC3, C3, and CP3) or right (FC4, C4, and CP4) cortex in half of all trials, respectively. This resulted in a total of four feedback sessions each of which lasted 12 min, i.e., regulating *left* hemisphere with *haptic* feedback, regulating *left* hemisphere with *visual* feedback, regulating *right* hemisphere with *haptic* feedback, regulating *right* hemisphere with *visual* feedback. For the classification of successful brain self-regulation an adaptive linear classifier procedure was used as described recently (Gharabaghi et al., 2014a; Vukelić et al., 2014; Vukelić and Gharabaghi, 2015). Each feedback session was subdivided into three runs with each run separated into 16 trials. To ensure that the participants remained in the deliberative phase of skill acquisition with high demands for learning self-regulation, we increased the task difficulty after each run, i.e., we increased the threshold value of the online classifier to ensure that feedback was provided only when the subjects reached either 50% (low difficulty), 30% (moderate difficulty), or 10% (high difficulty) of the strongest β-event-related desynchronization (ERD) modulation in the first, second and third run, respectively.

Before (PRE) and after (POST) the neurofeedback training, we recorded 6 min of resting state activity with the subjects alternating between the conditions “relax with *eyes open (EO)”* and “relax with *eyes closed (EC)” every* 15 s (Blankertz et al., 2010). During the EO condition, the subjects fixated a central cross on a computer screen. An auditory beep tone caused the subjects to switch between EC and EO conditions.

### Data Pre-Processing

The present analysis considered EEG data during the EO condition (Blankertz et al., 2010). Each 15 s period was concatenated, resulting in a data stream of 3 min per subject both for each PRE and POST neurofeedback recording. Artifacted EEG channels (PO9), that had been detected by visual inspection were not taken into account. The EEG data were detrended, zero-padded and band-pass filtered between 1–42 Hz, using a first order zero-phase lag Finite Impulsive Response (FIR) filter. We divided the whole data set into 3 s epochs, automatically rejecting any epochs that contained artifacts with an amplitude >100 μV (Sanei, 2007). Finally, the artifact-free EEG data was re-referenced to mathematically linked mastoids (Nunez, 2006).

### Estimation of β-Modulation Range

**Figure 1:**
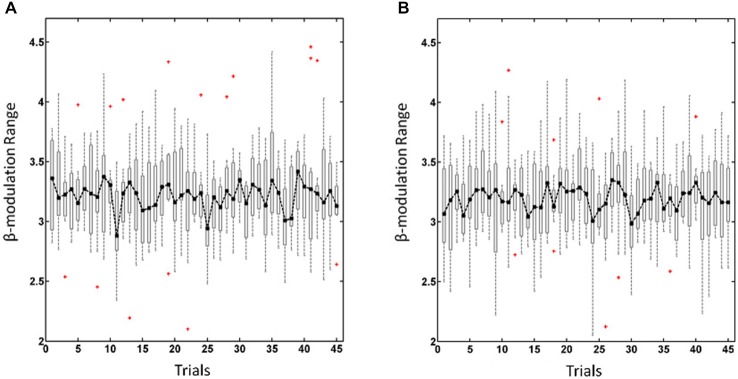
**β-modulation range for regulating the left hemisphere (A) and right hemisphere (B).** Dot-dashed line and boxplots show the median and the range, respectively, of the overall β-modulation, i.e., for the haptic and visual feedback sessions, across trials with red crosses indicating outliers.

To estimate the ability of each subjects to self-regulate his/her oscillatory activity during the training experiment we calculated the β-modulation range as described in detail elsewhere (Vukelić et al., 2014; Vukelić and Gharabaghi, 2015). In short, the β-modulation range was calculated off-line from the event-related spectral perturbation, as implemented in EEGLab toolbox (Delorme and Makeig, 2004), in the same frequency band (16–22 Hz) and from the same electrodes (FC3/4, C3/4, and CP3/4) as used for online control. This value described the maximal potential of each subject to synchronize and de-synchronize local oscillatory β-activity. The overall β-modulation range, i.e., for the haptic and visual feedback sessions, was calculated trial-wise for regulating the left (Figure 1A) and right hemisphere (Figure 1B) respectively. In cases where no trials had to be removed due to artifacts, we excluded the first trials in each run to adjust the number of trials in each subject. This resulted in the trials 1–15, 16–30, 31–45 for the first, second and third run, respectively (see Figure 1).

### Estimation of Functional Connectivity Networks

To calculate functional connectivity we utilized the imaginary part of coherence (iCOH; Nolte et al., 2004). iCOH is a robust connectivity measure ignoring relations at zero phase lag and is therefore insensitive to volume conduction properties. Since the original proposed iCOH might exhibit a spatial bias towards long-range synchronizations, we used the corrected version of the iCOH function (ciCOH) as suggested by Ewald et al. (2012). This version shares the same properties as the original iCOH function but includes additional features to compensate for the preference of remote interactions. ciCOH was calculated for each artifact free epoch, where the ciCOH function is based on an estimation of the complex coherency function. Hence, epochs were further divided into 1 s segments with 50% overlap resulting in a frequency resolution of *δf* = 1 Hz (Nolte et al., 2004). The segments were subsequently multiplied with a Hanning window, and the cross-spectrum between two time series, was defined by calculating the Fourier transformation and averaging over 1 s segments (Nolte et al., 2004):

(1) $$S_{ij}(f)=\frac{1}{N}\sum_{k-1}^{N}z_{i}(f,k)z_{j}^{*}(f,k)$$

where z*_i_*(·) and z*_j_*(·) represent the Fourier transform of the time series for channels *i* and *j*, *k* the segments of length 1 s, and *N* the total number of segments.

For each channel pair *i* and *j* the complex coherency function was defined as the normalized cross-spectrum:

(2) $$COH_{ij}(f)=\frac{S_{ij}(f)}{\sqrt{S_{ii}(f)S_{jj}(f)}}$$

Where *S_ij_*(·) was the cross-spectrum between channels *i* and *j*, and *S_ii_*(·), *S_jj_*(·) represented the auto-spectra for channels *i* and *j*, respectively.

Since, our neurofeedback training procedure focused on electrodes over selected sensorimotor regions, i.e., premotor (PM, FC3/4), primary motor (M1, C3/4) and primary somatosensory (S1, CP3/4) regions, we defined each of them separately as seed electrodes and evaluated systematically the functional connectivity between these circumscribed regions of interest (ROIs) and the whole brain (all other EEG channels). Hence, the ciCOH function was calculated from the complex coherency function (Ewald et al., 2012):

(3) ciCOHSeedj(f)=Im(COHSeedj(f))(1−Re(COHSeedj)2)

where *Seed* denotes the seed electrode *f* indicate frequency bins and *Im*(·) and *Re*(·) denote the imaginary and real parts, respectively. The ciCOH was fisher z-transformed to fit a Gaussian distribution (Rosenberg et al., 1989; Nolte et al., 2004). We evaluated the functional connectivity within predefined frequency bands of interest (FOI): α (8–14 Hz), low β (15–25 Hz), and high β (26–40 Hz). In a next step, the functional connectivity measure was obtained by averaging the absolute value of ciCOH across frequencies within each predefined FOI.

Furthermore, control analyses (i.e., control for spatial selectivity) of functional connectivity were conducted by defining seed electrodes immediately surrounding the neurofeedback ROIs, i.e., the electrodes adjacent to the FC3, C3, and CP3 electrodes, respectively. All data analysis was performed offline with custom written scripts in MATLAB®.

### Statistics

To analyze networks changes induced by brain self-regulation we compared the functional connectivity (ciCOH) between the PRE and POST condition. Here, we conducted a cluster-based permutation analysis which offers the opportunity to incorporate neurophysiologically motivated constraints to the test statistic (i.e., spatially clustering neighboring electrodes). This increases the sensitivity of the statistical test and controls for the family-wise error rate, thereby correcting for the multiple comparison problem (Nichols and Holmes, 2002; Maris and Oostenveld, 2007; Maris et al., 2007; Maris, 2012). This entailed the use of a cluster-based non-parametric randomization approach as implemented in FieldTrip (Oostenveld et al., 2011). Here, a multiple dependent sample t-statistic was conducted to establish the topography of resting state motor networks (i.e., seed electrodes) showing significant functional connectivity (ciCOH) differences between the POST and PRE training conditions for each predefined FOI. Thus, *t*-values exceeding a threshold of *p* < 0.01 (uncorrected) where spatially clustered based on neighboring electrodes. The cluster level statistics were defined as the sum of *t*-values within every cluster. The correction of multiple comparisons was carried out by considering the 95th percentile (two tailed) of the maximum values of summed *t*-values estimated from an empirical reference distribution. *t*-values exceeding this threshold were thus considered as significant at *p* < 0.05 (corrected).

The reference distribution of maximum values was obtained by means of a permutation test (randomly permuting the ciCOH across the POST and PRE training resting state EEG data for 1000 times). This non-parametric approach was used to evaluate the functional connectivity topographies of POST- vs. PRE-training differences of resting state brain activity.

## Results

The overall β-modulation, i.e., of the haptic and visual feedback sessions, were analyzed across trials for the left (Figure 1A) and right (Figure 1B) hemisphere, respectively, and revealed in a two-way ANOVA no main effects for “runs” *F*_(2,84)_ = 0.32, *p* = 0.72 or “hemisphere” *F*_(1,84)_ = 2.02, *p* = 0.16 nor for the interaction between these factors *F*_(2,84)_ = 0.27, *p* = 0.76. Thus, participants showed a stable performance of brain-self regulation for both hemispheres throughout the experiment, i.e., they adapted to the different levels of difficulty in each run.

**Figure 2:**
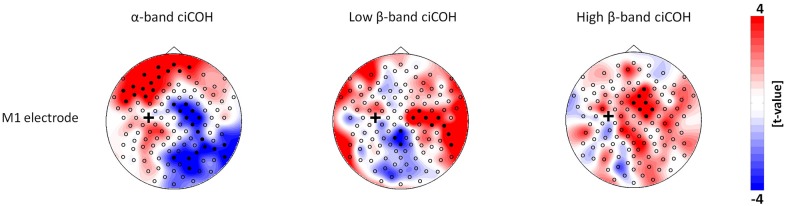
**Connectivity changes of the primary motor cortex.** The plots show *t*-value topographies of ciCOH for POST vs. PRE differences of intrinsic oscillatory activity in the α-band, low β-band, and high β-band. Electrode clusters, displaying significant differences in the non-parametric statistical randomization test, are visualized by filled black circles. The black cross indicates the seed electrode position in the primary motor cortex (M1). Red color indicates increase and blue color decrease in functional connectivity (ciCOH) in the POST training as compared to PRE training condition.

**Figure 3:**
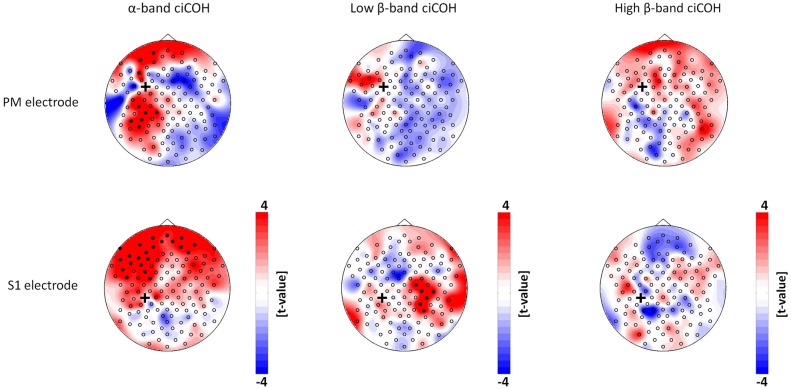
**Connectivity changes of the premotor and primary somatosensory cortex.** *t*-value topographies of ciCOH for POST vs. PRE differences of intrinsic oscillatory activity in the α-band, low β-band, and high β-band are shown. Electrode clusters, displaying significant differences in the non-parametric statistical randomization test, are visualized by filled black circles. The black cross indicates the seed electrode position in the premotor (PM) and primary somatosensory (S1) cortex. Red color indicates increase and blue color decrease in functional connectivity (ciCOH) in the POST training as compared to PRE training condition. Note that there were no significant differences for high β-band for both electrodes overlying somatosensory and premotor regions, and no significant differences for low β-band for the electrode overlying premotor regions.

**Figure 4:**
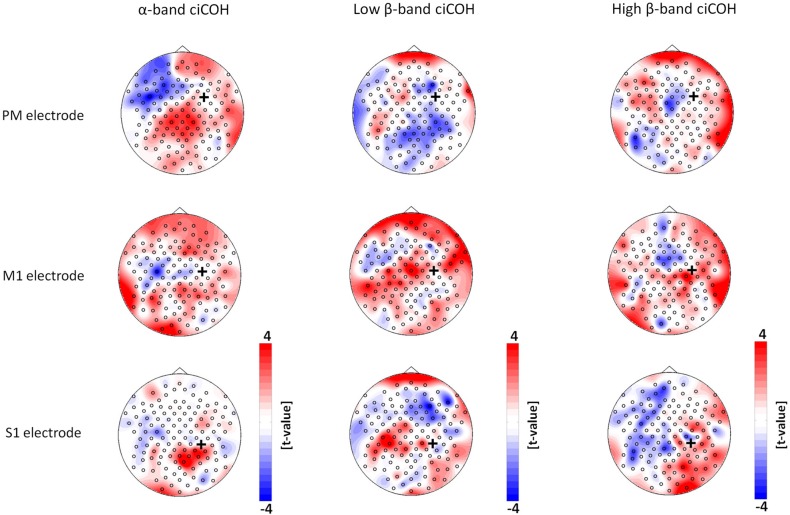
**Seed electrode analysis for the right hemisphere.** The plots show *t*-value topographies of ciCOH POST- vs. PRE-training differences with seed electrodes (marked by black cross) in premotor (PM, upper panel), primary motor (M1, middle panel), and primary somatosensory regions (S1, lower panel) of the right hemisphere. The first column shows the results for the α-band, the second column for the low β-band, while the third column shows the results for the high β-band. Note that no significant differences were observed.

**Figure 5:**
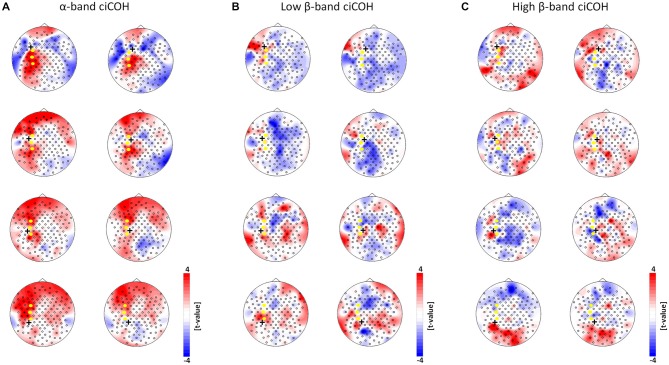
**Seed electrode control analyses of intrinsic functional network changes in the α-band (Panel A), low β-band (Panel B), and high β-band (Panel C).** The plots show *t*-value topographies of ciCOH POST- vs. PRE-training differences with seed electrodes (marked by black cross) immediately surrounding the targeted neurofeedback regions of interest (color coded in yellow). Red represents increase in functional connectivity; blue represents decrease in functional connectivity. Note that no systematic significant differences for the α-, low and high β-band were observed. Only two significant clusters in two different seed electrodes were observed in the α-band.

The non-parametric randomization test revealed significant changes of functional connectivity for the neurofeedback targets of the dominant left hemisphere (FC3, C3, CP3, see Figures 2, 3), but not of the non-dominant right hemisphere (FC4, C4, CP4, see Figure 4). These findings were spatially selective, i.e., they were not observed in the surrounding electrodes (see Figure 5). More specifically, we observed increased functional connectivity of both the seed electrodes overlying the primary motor and the primary somatosensory cortex of the left hemisphere with their respective contralateral homologous cortices in the low β-frequency band which were self-regulated during feedback (see Figures 2, 3, middle). Simultaneously, the seed electrode over the primary motor cortex presented a decrease of functional connectivity with electrodes in midline parietal area in the same frequency band (see Figure 2, middle).

At the same time, the seed electrode over the left primary motor cortex, and none of the surrounding electrodes in other cortical regions, exhibited an increased connectivity to contralateral electrodes over the supplementary motor and dorsal premotor areas in the high β-band (see Figure 2, right). For the α-band, all neurofeedback target ROIs of the left hemisphere showed an ipsilateral increase of functional connectivity with electrodes over frontal areas, while the electrode over the premotor cortex exhibited additional functional coupling with electrodes over parietal regions (see Figures 2, 3, left). Simultaneously, the seed electrode over the left primary motor cortex showed a decrease of functional coupling with electrodes in contralateral primary and secondary sensorimotor areas in the very same frequency band (see Figures 2, 3, left).

Neither the neurofeedback targets of the right hemisphere (FC4, C4, CP4, see Figure 4) nor any of the surrounding seed electrodes showed comparable changes of connectivity patterns (see Figure 5).

## Discussion

This study aimed to shed light on possible neurophysiological mechanisms of learning to volitionally modulate circumscribed brain activity by applying high-resolution electroencephalography to study the immediate after-effects of a single neurofeedback intervention on the resting state network architecture of oscillatory brain activity. While most previous studies exploring the influence of learning and neuroplastic changes on the subsequent intrinsic brain connectivity used functional magnectic resonance (Albert et al., 2009; Vahdat et al., 2011; Harmelech et al., 2013), we decided to instead use neuroelectrical recordings to enable us to examine frequency-specific measures of connectivity. The reason for this was that patterns of coherent oscillations have been shown to match with a broad variety of attentional, cognitive and sensorimotor behavior (Destexhe et al., 1999; Steriade, 2006; Engel and Fries, 2010; Siegel et al., 2012; Engel et al., 2013). For the purpose of restoring lost motor functions for example, neurofeedback of sensorimotor β-band (15–30 Hz) activity seems to be particularly suited (Gharabaghi et al., 2014a,b,c) as this frequency band is linked to the natural communication between cortex and peripheral muscular activity. However, even these approaches have been shown to activate a distributed cortical network in a lower, i.e., α-frequency band (Vukelić et al., 2014), thereby bridging the abilities and cortical networks of motor imagery and motor execution (Bauer et al., 2015).

Previous studies on visuomotor skill learning suggested that the same networks which connected prefrontal cortices (PFC), premotor (PM) regions, supplementary motor areas (SMA), primary sensorimotor, and parietal cortices, and which were recruited in the course of training, shaped the pattern of the following intrinsic brain activity (Albert et al., 2009; Vahdat et al., 2011). These resting state patterns would therefore reflect the history of neuronal activation during the skill learning period encompassed as lasting increases and/or decreases of connectivity among these cortical regions. Such neuronal changes have been shown to involve both short-term (immediate) and long term (long-lasting) Hebbian-like effects of previous cortical activation (Harmelech et al., 2013). This very study was the first to describe such effects on intrinsic networks following brain self-regulation via functional Magnetic Resonance maging (MRI)-based neurofeedback. However, due to the nature of the technique applied, the co-activations of distant cortical areas could not be characterized on different frequency scales. Here, we successfully extended this line of research by using EEG as a tool to capture frequency-specific measures of functional connectivity.

By regularly switching the feedback modality and the trained cortical hemisphere, and by continuously increasing the difficulty of the feedback task, we succeeded in keeping the subjects in the deliberative phase of skill acquisition throughout the whole experiment to trace learning and not performance related connectivity changes in the subsequent intrinsic networks.

Switching between feedback modalities might have caused the overall effects of the intervention to be determined by the characteristic features of only one modality, e.g., the sensory stimulation of the haptic feedback. A recent study addressed this question by contrasting the very same two feedback modalities as in the present study while capturing the entrained cortical networks during the task (Vukelić and Gharabaghi, 2015). This comparison between haptic/proprioceptive and visual feedback revealed, with respect to the same frequency spectrum analyzed in the present study, significant differences only for the low β-band. In this low β-frequency band, the haptic condition revealed a significantly stronger decoupling of the trained, i.e., left, motor cortex from bilateral premotor and frontal areas as compared to the visual feedback condition, i.e., a pattern relevantly different from those connectivity changes observed in the present study. It is therefore plausible to assume that the findings of the current study were not determined by one feedback modality only.

In this context, it is remarkable that the learning-related connectivity changes were lateralized to the dominant left hemisphere of the participants despite the fact that both hemispheres underwent the same amount of feedback training. This observation might reflect the functional specialization, i.e., that planning of manual actions of either hand involves the left posterior parietal and the left motor area (Rushworth et al., 2003; Johnson-Frey et al., 2005; Bauer et al., 2015). Similarly, the ability to execute movements of the left hand is also characterized by connectivity within bilateral motor regions, especially by signals from the left to the right motor areas. This might reflect the relay of planned movements from the left to the right hemisphere, in accordance with hemispheric specialization in right-handers (van den Berg et al., 2011; Bauer et al., 2015). Interestingly enough, we were able to demonstrate how these interhemispheric communications were mediated in different frequency bands in a complex way, i.e., increased functional connectivity of the seed electrode overlying the primary motor cortex of the left hemisphere with its contralateral homologous cortex in the low β-frequency and with contralateral electrodes over the supplementary motor and dorsal premotor areas in the high β-band. At the same time, there was a decrease of functional connectivity of the very same seed electrode over the primary motor cortex with contralateral electrodes over primary and secondary sensorimotor areas in the α-band and with electrodes over midline parietal area in the low β-band.

Bilateral somatomotor regions are known to have a high inclination to oscillate synchronously in the β-band during intrinsic natural brain activity (Marzetti et al., 2013). The stronger engagement of interhemispheric sensorimotor cortices might represent an initial recruitment of homologues regions, where the interplay of these interactions undergoes dynamic plastic changes. This is crucial for motor control and motor skill learning (Beaulé et al., 2012; Takeuchi et al., 2012). With regard to brain lesions, such as occur following a stroke, maladaptive neuronal reorganization of interhemispheric primary sensorimotor cortices are related to impaired motor and cognitive behavior (Rehme et al., 2011a,b; Dubovik et al., 2012). Furthermore, abnormal alterations of intrinsic functional communication between the sensorimotor network and higher order supplementary motor cortex is also related to impaired motor behavior after stroke (Inman et al., 2012). It is worth mentioning that we also detected frequency (high β-band) specific effects of increased communication between the electrode over the left primary motor network connected with higher order motor regions such as electrodes over the SMA and dorsal PM regions in the right hemisphere (Figure 2). This could be due to the fact that already motor skill learning involves two parallel cortico-subcortical-cerebellar circuits (frontoparietal striatum-cerebellar loop and sensorimotor striatum-cerebellar loop) which coordinate both spatial and motor features of learning (Hikosaka et al., 2002). In this context, the communication between primary motor, SMA, and PM regions is liable to coordinate the transformation between these two systems related to different aspects of skill acquisition (Hikosaka et al., 2002; Vahdat et al., 2011). Recent results showing a higher recruitment of SMA in relation to positive brain-computer interface (BCI) control (Halder et al., 2011) are also in agreement with our observation and further highlights the special relevance of SMA for neurofeedback training.

Synchronization of oscillations in the α-band has been proposed to underlie attentional states, memory processes and motor planning during sensorimotor behavior (Sauseng and Klimesch, 2008; Palva and Palva, 2011; Siegel et al., 2012). Our results highlight an immediate after-effect of neurofeedback training on distributed fronto-centro-parietal networks synchronously oscillating in the α-band (Figures 2, 3). We found a consistent increase of the functional connections between electrodes over the left PFC with electrodes over the left PM and primary sensorimotor regions. Left PM regions and PFC are primarily involved in the skill acquisition of new motor sequences and in the short-term storage and encoding of these new learned sequences (Schubotz and von Cramon, 2003; Hardwick et al., 2013). Increased functional connectivity between PFC and PM are probably related to high attentional demands (Hikosaka et al., 2002; Sun et al., 2007) during skill learning. The increased connections between PFC and primary sensorimotor regions could therefore be related to short-term storage of information and encoding of unfamiliar new skills and could also reflect higher cognitive and attentional demands (Grafton et al., 2008; Kantak et al., 2012). Moreover, we found a dissociation of interhemispheric communication between electrodes in bilateral primary sensorimotor regions in the α- and β-band (Figure 2). The α-band showed a down-regulation, while the β-band showed an up-regulation of functional connectivity. These results possibly reflect a cross-frequency interaction between these two components, which has frequently been observed during cognitive tasks (Palva et al., 2005) and which has also been found to be present during intrinsic brain oscillations (Nikulin and Brismar, 2006; Chella et al., 2014). Along these lines, we recently demonstrated that synchronized coupling of global α-oscillations regulated the volitional modulation of regional β-band sensorimotor activity related to the successful control of these oscillations (Vukelić et al., 2014). In such a cross-frequency framework, a hierarchy seems to exist in which the lower frequencies modulate the oscillations of higher frequencies (Jensen and Colgin, 2007; Canolty and Knight, 2010).

From the methodological point of view, brain connectivity analysis has to disentangle *true* neuronal interferences from the phenomenon of *volume conduction* or *field spread*, which occurs at zero time (or phase) lag (Nolte et al., 2004; Stam et al., 2007; Schoffelen and Gross, 2009; Ewald et al., 2012). However, *true* neuronal activity measured with EEG might show zero (or close to zero) time lag at local or distant cortical regions as well (Stam et al., 2007; Schoffelen and Gross, 2009; Ewald et al., 2012). Both empirical data (Roelfsema et al., 1997) and modeling findings (Vicente et al., 2008), have revealed symmetrical interaction, i.e., in phase or in phase opposition, among distant neuronal populations. This common source problem might affect connectivity measures such as the *classical* iCOH (Stam et al., 2007; Vinck et al., 2011; Ewald et al., 2012). The *corrected* form of the iCOH function (ciCOH), which is used in the present study, intends to address this challenge by maximizing the imaginary part of the complex cross-spectrum (Ewald et al., 2012).

Furthermore, the present study has certain limitations with regard to the localization of coherent effects among distant cortical regions. The anatomical relationship between EEG potentials from surface electrodes and specific cortical structures is unsatisfactory due to the field spread effect of neuronal signals recorded at scalp EEG electrodes. However, the signals obtained are highly weighted by the proximity and radial orientation of the cortical area under the electrode (Nunez, 2006). Furthermore, it has been demonstrated that high resolution electrode systems with up to 128 channels, such as was used in this study, facilitates the spatial resolution more significantly than standard low resolution systems (32 and 64 electrodes; Luu et al., 2001). The use of the ciCOH function improves the spatial specificity further when connectivity is studied among EEG sensors. This diminishes the tendency to favor long-range interactions, thus also highlighting short-range interactions that would remain hidden (Ewald et al., 2012). One possible way of improving spatial specificity among scalp related EEG potentials could be the use of surface laplacian. However, it is important to note that such a transformation could unintentionally distort phase synchronization effects due to distortions of physiologically generated phase differences, thereby precluding meaningful physiological results (Nunez, 2006).

In conclusion, our results demonstrated that a single neurofeedback intervention suffices to induce immediate reorganization of neuronal communications involving functional connectivity of frequency specific networks indicative for short term Hebbian-like processes.

## Conflict of Interest Statement

The authors declare that the research was conducted in the absence of any commercial or financial relationships that could be construed as a potential conflict of interest.
